# When Is Replacement Feeding Safe for Infants of HIV-Infected Women?

**DOI:** 10.1371/journal.pmed.0040030

**Published:** 2007-01-16

**Authors:** Grace C John-Stewart

In this issue of *PLoS Medicine*, Renaud Becquet and colleagues report their findings from a new study looking at the long-term safety of infant feeding interventions aimed at reducing mother-to-child HIV transmission in Africa [1]. Over two years, the researchers studied the safety of infant feeding interventions (either formula feeding or shortened breast-feeding) among infants of HIV-infected mothers in Abidjan, Côte d'Ivoire.

## The UNAIDS Guidelines

The authors chose to examine this issue because of the continued challenges faced by HIV-infected mothers regarding infant feeding. Breast-milk transmission of HIV contributes substantially to the risk of infant HIV infection; consequently HIV-infected mothers in Europe and the United States are counseled not to breast-feed their infants. However, avoiding breast-feeding or shortening the term of breast-feeding may be risky for infants in settings with inadequate sanitation, limited access to breast-milk substitutes, or unsafe water. Thus, UNAIDS (the Joint United Nations Programme on HIV/AIDS) recommends that HIV-infected women “replacement feed” their infants when it is acceptable, feasible, affordable, sustainable, and safe (“replacement feeding” is a term used to refer to feeding infants with milk other than breast milk, such as formula) [2].

Interpreting these guidelines is particularly challenging on the ground. When is replacement feeding safe for babies? This issue of safety is the focus of Becquet et al's study—by measuring severe morbidity and mortality, the authors determined how risky replacement feeding and shortened breast-feeding were for infants born to HIV-infected mothers in an urban African setting.

## The New Study

The authors compared morbidity, hospitalization, and mortality over a two-year period among infants of HIV-infected women who had received peripartum antiretrovirals and elected to either breast-feed for four months or formula feed. In addition, the authors compared the infants in this cohort to infants in a historical cohort in the same setting with the same clinical and survival assessments who breast-fed for a prolonged duration.

Breast-milk transmission of HIV contributes substantially to the risk of infant HIV infection.

Becquet et al. found that at baseline, formula-feeding mothers were more educated and had better access to tap water and less shared housing than women who elected to breast-feed for a short duration. However, there was no evidence that formula-feeding mothers in this cohort had more advanced HIV disease than women who breast-fed. Overall, adverse events among HIV-uninfected infants were similar in the two groups. Formula-fed infants had significantly increased risk of diarrhea and acute respiratory illnesses, while short-term breast-fed infants tended to have a higher incidence of malnutrition. However, over two-year follow-up, the risk of hospitalization or mortality did not differ between the two feeding groups.

In addition, when both groups of infants were compared to a historical cohort in which breast-feeding was prolonged, the authors found excellent two-year survival among HIV-uninfected children regardless of whether they breast-fed long-term (95%) or breast-fed for four months or never breast-fed (96%). The authors conclude that “given appropriate nutritional counseling and care, access to clean water, and a supply of breast-milk substitutes, these alternatives to prolonged breast-feeding can be safe interventions to prevent mother-to-child transmission of HIV in urban African settings.”

## Implications of the Study

These conclusions may be slightly provocative in 2007—replacement feeding has been abandoned in many African settings as a viable intervention for prevention of breast-milk HIV transmission. The authors provide good data to suggest that with appropriate provisos, replacement feeding can be a safe option to consider for HIV-infected mothers in urban African settings. Are these conclusions valid? Yes. The study has several strengths—the authors have conducted meticulous and robust analyses with large cohorts including a long period of follow-up. The authors had adequate retention and detailed morbidity assessment, and they included independent assessment of morbidity. The authors made use of a closely linked historical cohort in order to compare prolonged breast-feeding with shortened or no breast-feeding. While exclusive breast-feeding was not practiced by the majority of women in the cohort despite counseling to do so, the proportion of predominantly breast-feeding women was similar to other African cohorts promoting exclusive breast-feeding and probably reflects the reality of breast-feeding practices in similar settings [3]. All in all, for a woman in Abidjan, the likelihood of having a baby neither acquire HIV nor die would be highest with peripartum antiretrovirals and no or limited breast-feeding.

Are the conclusions generalizable? Perhaps not widely. The findings of this study are consistent with a clinical trial in Nairobi, in which infants of HIV-infected women randomized to formula feed had significantly higher likelihood of not having an adverse outcome (either death or HIV infection) than the infants of women randomized to breast-feed [4]. Unfortunately, both studies may be hard to generalize to diverse settings in Africa. Supplying breast-milk substitutes is possible, but ensuring clean water is more problematic. In addition, the very strengths of the study (i.e., systematic frequent follow-up to ascertain morbidity and mortality) provided a safety net to prevent morbidity and mortality due to not breast-feeding that may be hard to replicate in a busy maternal–child health clinic in other settings. Thus, some prevention of mother-to-child transmission of HIV (PMTCT) programs that have provided replacement feeding have noted increased hospitalizations among infants who were replacement fed [5]. In addition, policy makers remain concerned about the potential impact of replacement feeding spillover from PMTCT programs to the general community, which could undermine efforts to promote breast-feeding [6]. Overall, translating safety for infant replacement feeding from closely observed research cohorts to programs can be problematic.

The minimal mortality observed by Becquet and colleagues, however, may motivate PMTCT programs to think more carefully about optimizing child health in general. The high two-year survival in this cohort is laudable and reflects an important goal for PMTCT programs that may currently neglect child survival strategies in a narrow focus on HIV prevention. Incorporating frequent follow-up, growth assessment, and morbidity assessment and treatment within PMTCT follow-up clinics is essential to preserve child health benefits in addition to preventing infant HIV infection. While water safety and confidentiality issues may still pose challenges, strengthened infrastructure for infant/child surveillance may contribute to making replacement or shortened breast-feeding more safe.

## Conclusion

Current research in prevention of breast-milk HIV transmission includes evaluation of a variety of approaches, including optimizing exclusive breast-feeding, providing antiretrovirals during shortened breast-feeding to mother or infant, and, ultimately, vaccination. Preserving breast-feeding is attractive because of nutritional, growth, safety, and confidentiality issues, and in the future these approaches may enable prolonged breast-feeding. However, some of the interventions currently under study may not be as promising as initially envisioned. For example, highly active antiretroviral therapy (HAART) during shortened breast-feeding is not the panacea hoped for—it may be associated with loss of confidentiality, toxicity, resistance, and infant morbidity and growth compromise when breast-feeding is stopped at six months. It is plausible that strategizing for not breast-feeding from birth would be less problematic than first starting to breast-feed on HAART, and then stopping after six months. Thus, replacement feeding should still be considered in the mix of strategies to prevent breast-milk transmission of HIV, particularly when water safety is assured and provision of breast-milk substitutes is an option. Becquet et al's data are reassuring that when appropriate support is provided and clean water is available, replacement feeding can be safe in an urban African setting.

## Abbreviations

HAART: highly active antiretroviral therapy
PMTCT: prevention of mother-to-child transmission of HIV
